# An Appraisal of Developments in Allium Sulfur Chemistry: Expanding the Pharmacopeia of Garlic

**DOI:** 10.3390/molecules24214006

**Published:** 2019-11-05

**Authors:** Peter Rose, Philip Keith Moore, Matthew Whiteman, Yi-Zhun Zhu

**Affiliations:** 1 School of Biosciences, University of Nottingham, Loughborough, Leicestershire LE12 5RD, UK; 2 School of Pharmacy and State key Lab. of Quality Research in Chinese Medicine, Macau University of Science and Technology, Macau, China; yzzhu@must.edu.mo; 3 Department of Pharmacology, Yong Loo Lin School of Medicine, National University of Singapore, Singapore 117597, Singapore; dprmpk@nus.edu.sg; 4 Medical School Building, St Luke’s Campus, Magdalen Road, Exeter EX1 2LU, UK; M.Whiteman@exeter.ac.uk

**Keywords:** allium, anticancer, sulfur compounds, gaseous mediators, hydrogen sulfide, garlic

## Abstract

Alliums and allied plant species are rich sources of sulfur compounds that have effects on vascular homeostasis and the control of metabolic systems linked to nutrient metabolism in mammals. In view of the multiple biological effects ascribed to these sulfur molecules, researchers are now using these compounds as inspiration for the synthesis and development of novel sulfur-based therapeutics. This research has led to the chemical synthesis and biological assessment of a diverse array of sulfur compounds representative of derivatives of *S*-alkenyl-l-cysteine sulfoxides, thiosulfinates, ajoene molecules, sulfides, and *S*-allylcysteine. Many of these synthetic derivatives have potent antimicrobial and anticancer properties when tested in preclinical models of disease. Therefore, the current review provides an overview of advances in the development and biological assessment of synthetic analogs of allium-derived sulfur compounds.

## 1. Introduction

Approximately 700 plant species are known to contain *S*-alk(en)yl-l-cysteine sulfoxides (ACSOs), including many edibles like allium and brassica vegetables (Table 1). Popularity stems from their distinctive flavors, produced during the breakdown of each respective ACSOs [1,2]. As such, changes to the composition and quantities of each ACSO determines the odor, flavor variation, and biological activities ascribed to many plants containing these sulfur storage compounds [3]. At present, four major and two minor ACSOs exist, and these act as progenitor molecules in the production of other sulfur compounds like the lachrymatory factor, propanethial S-oxide and the antimicrobial compound allicin [3]. An additional 50+ sulfur compounds, including thiosulfinates, cysteine, and sulfides derivatives can also occur in allium tissue following preparation, and many of these compounds possess some level of biological activity in mammalian cells and tissue [4]. Indeed, researchers have shown crude allium-derived plant extracts as well as isolated sulfur compounds such as *S*-allylcysteine, diallyl disulfide, allicin, 1,2-vinyldithiin, 3-vinyldithiin, and ajoene can induce a range of physiological and biochemical changes in mammalian systems [5]. For example, several cellular targets are reported to be susceptible to sulfur compound treatment, including proinflammatory transcription factor nuclear factor-kappa beta (NF-κB) [6,7], various kinases, including p38 mitogen-activated protein kinase (p38 MAPK) [8], c-JunNH2-terminal kinase (JNK) [9], extracellular signal-regulated kinase (ERK) [10], and phosphoinositide 3-kinase-protein kinase B (PI-3K-Akt) [11]. Other cellular proteins, like nuclear factor erythroid 2-related factor 2 (Nrf-2) [12,13,14], p53 [15,16], AMP-activated protein kinase [17,18], proliferator-activated receptor γ [19], and NAD-dependent deacetylase sirtuin-1 (SIRT1) [20] are also targets of allium-derived sulfur species. Consequently, many sulfur molecules have been reported to have anticancer properties [21,22,23,24], are anti-inflammatory [7,25,26,27], act as cytoprotective agents [28,29], function as inhibitors of metastasis [30,31], and alter lipid metabolism [32,33]. Additionally, molecules like diallyl trisulfide (DATS) release hydrogen sulfide gas (H_2_S) in the presence of glutathione and cysteine [34]. The liberated H_2_S acts as a gaseous signaling molecule in mammalian cells with potential pharmacological effects in humans [35,36]. This property may contribute to the biological effects attributed to DATS and, more widely, other allium-derived sulfur species [37]. The identification of these molecular targets may explain why several ACSOs containing plant species, including garlic (*Allium sativum)* and onion (*A. cepa)* are common constituents of folklore remedies [38,39,40]. Moreover, several human population-based studies have shown that the consumption of diets rich in allium and brassica vegetables are associated with a reduced risk of developing several forms of cancers [41,42], type II diabetes [43], cardiovascular diseases [44,45], and changes in the gut microbiome [46]. 

In light of the potential roles of dietary sulfur compounds and their potential impact on health, it is timely to address some of the lesser-known aspects relating to research on allium-derived sulfur compounds, that of the biological screening of synthetic analogs of ACSOs and associated degradation products. Over the last decade, a number of researchers have synthesized a spectrum of synthetic sulfur compounds, representative of derivatives of thiosulfinates, cysteine analogs, ajoene-based compounds, and various sulfides. Sulfur-derived functional groups occur in a broad range of pharmaceuticals and natural products, and facilitated developments in modern drug design [47]. In the case of allium-based molecules, some are reportedly superior to their natural counterparts, and could be useful in the future development and design of new sulfur therapeutics, or as tools to explore sulfur metabolic networks in living organisms. Therefore, the current review describes recent advances in the biological assessment of some novel sulfur molecules that have been inspired by allium sulfur chemistry.

## 2. Allium Sulfur Storage Compounds and Generation of Biologically Active Compounds

**Figure 1:**
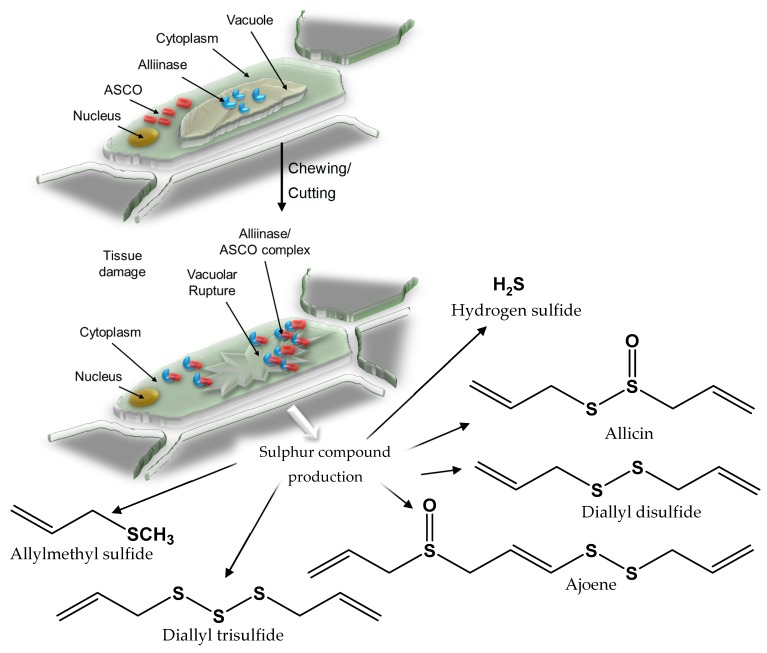
Catabolism of *S*-alk(en)yl-l-cysteine sulfoxides (ASCOs) by alliinase enzyme generates a spectrum of individual sulfur compounds.

For the purposes of this review, only a brief overview of the types of sulfur compounds commonly encountered in allium and brassica plants is provided. We refer interested readers elsewhere for additional coverage of allium sulfur biochemistry [1,2,3,4,5]. In brief, the main parental storage compounds in alliums are (+)-*S*-allyl-l-cysteine sulfoxide (S-ACSO), commonly known as alliin, (+)-*S*-methyl-l-cysteine sulfoxide (methiin; MCSO), (+)-*S*-propyl-l-cysteine sulfoxide (propiin; PCSO), and (+)-*S*-trans-1-propenyl-l-cysteine sulfoxide or isoalliin (TPCSO) [1,2]. Isoalliin is the major sulfoxide present within intact onion tissue and is the source of the lachrymatory factor, thiopropanal-*S*-oxide. (+)-*S*-methyl-l-cysteine sulfoxide is by far the most ubiquitous, found in varying amounts in the intact tissue of *A. sativum, A. cepa, A. porrum*, and *A. ursinum* L., as well as in some brassica vegetables (Table 1). Enzymatic hydrolysis of ACSOs by the enzyme alliinase (EC 4.4.1.4) produces pyruvate, ammonia, and sulfenic acids [48]. Formed sulfenic acids further condense to form thiosulfinates, which in turn form a spectrum of additional sulfur compounds (Figure 1). These sulfur compounds are believed to be partly responsible for the reported health benefits attributed to the consumption of an allium- and brassica-rich diet [38,40,41,42,43,44,45,46]. Less widely reported are advances in the synthesis and biological assessment of allium-based sulfur analogs.

## 3. New Sulfur Compounds Inspired by Allium Metabolites

In recent times, a number of researchers have focused their attention on the development of novel synthetic sulfur analogs of known allium-derived sulfur compounds. These molecules were conceptualized with the view to 1) produce more stable chemical entities with enhanced solubility; 2) develop molecules with specific chemical properties, like the ability to release H_2_S or nitric oxide (NO) gas; or 3) possess altered biological activities, such as enhanced anticancer activities, interesting antioxidant properties, or antimicrobial effects. At present, the chemical derivatives of alliin, allicin, *S*-allylcysteine, and garlic-derived sulfides have been reported in the literature. Of these, only a handful have been evaluated in preclinical models of disease and, as far as we are aware, none tested on humans (Table S1). Given the resurgent interest in sulfur biochemistry, it is reasonable to discuss advances in this area, as there is optimism that future research in this field will lead to the development of newer sulfur therapeutics. 

### 3.1. S-Alk(en)yl-l-cysteine Sulfoxides and Thiosulfinate Analogs

To date, only a handful of studies have focused on the synthesis and evaluation of ASCO analogs. In 1985, the alliin molecule was isolated from the tissue of *A. cepa,* and found to inhibit platelet aggregation [49]. Further work led to the testing of several commercially available alliin derivatives, and to the identification of S-oxodiallyl disulfide that reportedly inhibits platelet aggregation at similar concentrations as alliin [50]. ASCO catabolism leads to the formation of thiosulfinates, and this chemical group has gained considerable interest over the years. Thiosulfinates react with thiol groups in cells, and this reaction mechanism is responsible for their antimicrobial and antifungal activities [2,51,52]. The electron-withdrawing effect of the oxygen atom generates an electrophilic sulfur center, which can react with thiolate ions of small-molecular-weight compounds or thiol-containing enzymes. Despite the potential of using the thiosulfinate allicin as an antimicrobial agent, it is unstable and readily decomposes or binds to cellular proteins [53,54]. Indeed, when allicin is maintained at 20 °C for 20 h, it decomposed to diallyl disulfide (DADS) (66%), diallyl sulfide (DAS) (14%), diallyl trisulfide (DATS) (9%), and sulfur dioxide (SO_2_) [55]. For this reason, the development of thiosulfinate-based therapeutics has been limited. Newer fluorinated and *S*-aryl alkylthiolsulfinate analogs appear to have greater chemical stability, and these molecules may be promising in the future developments of thiosulfinate-based therapeutics [56,57]. 

**Figure 2:**
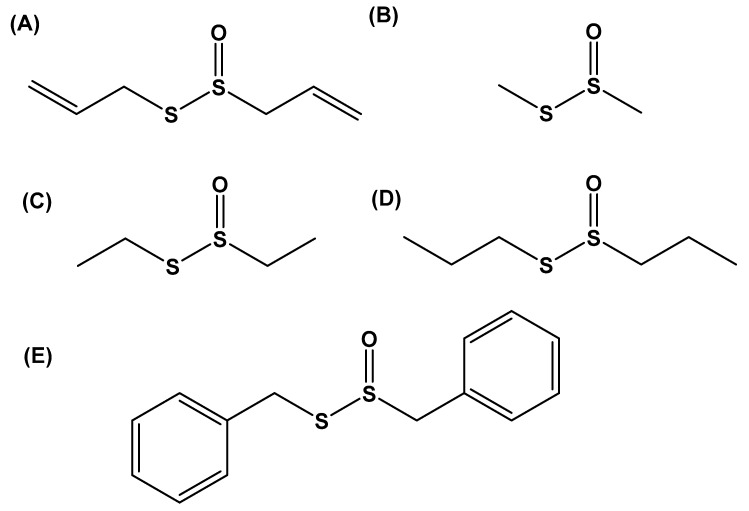
Structures of novel antimicrobial and antifungal thiosulfinate derivatives of (**A**) allicin, (**B**) dimethylthiosulfinate, (**C**) diethylthiosulfinate, (**D**) dipropylthiosulfinate, and (**E**) dibenzylthiosulfinate (adapted from [61])**.**

Recently, researchers have indeed raised the possibility of designing thiosulfinate-based prodrugs [58]. In this work, methionine- and cysteine-based compounds were tested. These molecules required catabolism by the methionine γ-lyase enzyme (MGL, EC 4.4.1.11) in order to generate reactive thiosulfinate intermediates. MGL can catalyze the γ- and β-elimination of methionine, and the cysteine analogs of l-methionine, l-methionine sulfoxide, *S*-ethyl-l-homocysteine, *S*-ethyl-l-homocysteine sulfoxide, *O*-acetyl-l-homoserine, *S*-ethyl-l-cysteine, *S*-ethyl-l-cysteine sulfoxide, and *O*-acetyl-l-serine and alliin. Of the tested compounds, l-methionine sulfoxide, alliin, *S*-ethyl-l-cysteine, and *S*-ethyl-l-homocysteine had bacteriostatic effects towards *Citrobacter freundii* and *Staphylococcus aureus* [58]. Similarly, Leontiev et al. focused on the synthesis of several antimicrobial analogs of allicin [59]; these compounds were representative of dimethyl-, diethyl-, diallyl-, allicin, dipropyl-, and dibenzyl- thiosulfinate derivatives (Figure 2A–E). In tests, MICs for the tested bacteria were in the range of 8–256 µg mL^−1^ and MBCs in the range of 16–256 µg mL^−1^ for various thiosulfinates. DMTS was more active against *P. fluorescens* than allicin was.

**Figure 3:**
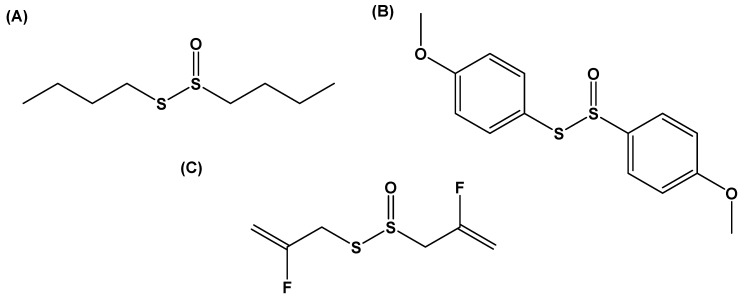
Structures of novel synthetic sulfur compounds based on the naturally occurring thiosulfinate allicin. Several thiosulfinate derivatives have anticancer properties, like (**A**) *S*-butyl butane-1-sulfinothioate, (**B**) *S*-4-methoxyphenyl 4-methoxybenzenesulfinothioate [60], and (**C**) novel antithrombotic agent difluoroallicin [61].

Other researchers have explored the use of thiosulfinates as novel anticancer agents. In the work of Roseblade et al., the synthesis and biological assessment of a series of 22 aromatic and aliphatic thiosulfinates were determined. These compounds were screened in an in vitro breast-cancer model using human adenocarcinoma breast-cancer cell lines MCF-7 and multidrug resistant (MDR) subline MCF-7/Dx [60]. Respective synthetic analogs *S*-butyl butane-1-sulfinothioate and *S*-4-methoxyphenyl-4-methoxybenzenesulfinothioate (Figure 3A,B) were identified as lead anticancer agents, having greater potency than that of allicin.

Of the tested aromatic thiosulfinates, anticancer activity was correlated with the substituent attached to the sulfenyl sulfur moiety. Those molecules have an electron-rich 4-methoxybenzyl group and are anti-proliferative in mammalian cells. Further assessment of S-4-methoxyphenyl-4-methoxybenzenesulfinothioate showed this compound to inhibit cell proliferation by inducing G2/M phase cell-cycle arrest and apoptosis in drug-resistant cells. Finally, the synthesis, reactivity, and anti-thrombotic and anti-angiogenesis activity of a fluorinated allicin derivative have recently been tested [61]. Difluoroallicin (*S*-(2-fluoroallyl) 2-fluoroprop-2-ene-1-sulfinothioate; Figure 3C) dose-dependently inhibited angiogenesis and suppressed platelet aggregation [61].

### 3.2. Ajoene Derivatives

**Figure 4:**
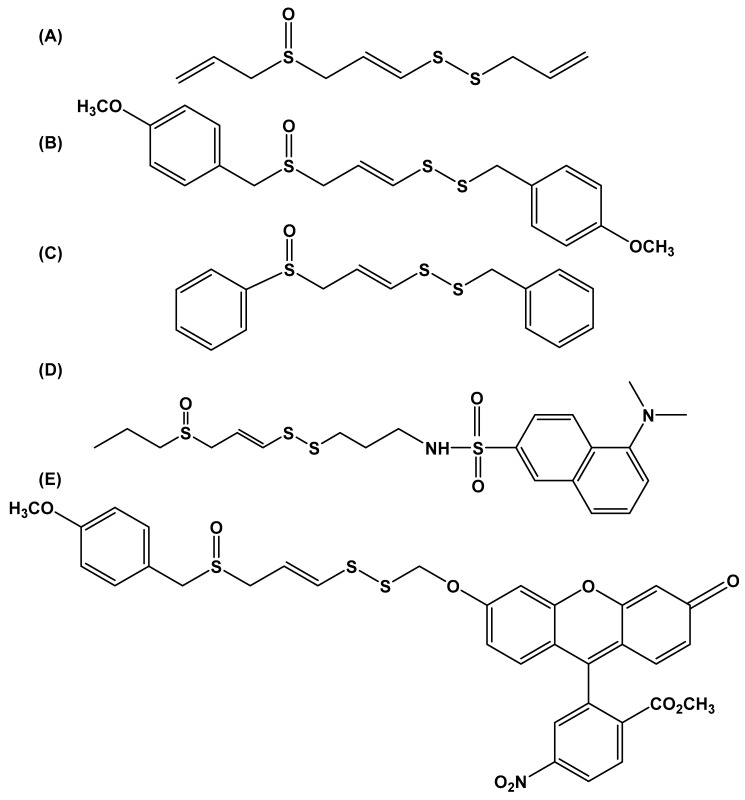
(**A**) Ajoene has inspired derivatives synthesis like (**B**) *(E,Z*)-1,8-(*Bis-para*-methoxyphenyl)-2,3,7-trithia-octa-4-ene [73], and (**C**) SPA3015 [74], molecules with anti-proliferative effects in cancer cells. Other studies have produced (**D**) a labelled dansyl and (**E**) fluorescein ajoene derivatives to assist in the identification of molecular targets prone to ajoene modification in mammalian cells [75,76].

Ajoene (Figure 4A) is a common component of oil macerates of garlic and is present in aged crushed-garlic preparations [62]. The vinyl sulfur atom located within its disulfide backbone is electrophilic [63] and reacts with nucleophilic thiols groups. Indeed, past work has shown ajoene to react with cysteine residues present in and on proteins such as in glutathione reductase [64], trypanothione reductase [64], and human gastric lipase [65]. The ability of this molecule to react with thiol residues is partly responsible for its antimicrobial and anticancer properties [63,66,67]. Several ajoene-based analogs retain the central vinyl disulfide/sulfoxide core but have a variable terminal end group. Recent studies indicate that the antimicrobial properties of ajoene can be partly explained by its ability to disrupt quorum sensing (QS) in bacteria like in the pathogenic bacterium *Pseudomonas aeruginosa* via reducing the expression of key virulence genes [68]. This property suggests that ajoene and associated analogs could be useful antimicrobial agents. To date, one structure–activity relationship (SAR) screened 25 disulfide bond-containing ajoene analogs [69]. Synthesis started with the replacement of the allyl group with differing aliphatic and aromatic groups coupled with the retention of the benzothiazole disulfide moiety. This work lead to the discovery of a number of active antimicrobial agents, all of which required the presence of the benzothiazole group for activity. *Para*-chlorophenyl derivative 2-((4-chlorophenyl)disulfanyl)benzothiazole significantly reduced the levels of QS-regulated virulence factors like elastase, rhamnolipid, and pyocyanin and inhibited *P. aeruginosa* infection in a murine model of implant-associated infection [69]. 

Some ajoene derivatives act as anticancer agents [70]. As shown in Table S1, a number of ajoene derivatives containing the central vinyl disulfide/sulfoxide core are cytotoxic to cancer cells. The *para*-methoxybenzyl (*bis*PMB) molecule (Figure 4B) and associated derivatives are anti-proliferative when tested in transformed CT-1 fibroblast cells. These same compounds also inhibited cancer-cell proliferation when tested on cultured prostrate, breast, cervical, and esophageal cancer cells [71,72]. Interestingly, analogs lacking the disulfide bond were inactive, indicating that the vinyl sulfur atom is important for biological activity. Mechanistic work indicates that *bis*PMB induces alternate splicing of transcription factor XBP-1 and alters the expression of ER stress proteins GRP78 and CHOP/GADD153, CHOP expression being central to the cytotoxicity of this molecule [73]. Other research has led to the identification and characterization of another ajoene derivative, SPA3015 (Figure 4C). SPA3015 reduces cell viability in P-gp-overexpressing MDR cancer cells, and suppresses NF-κB signaling. The inhibition of NF-κB decreases expression of anti-apoptotic proteins, the cellular inhibitor of apoptosis protein-1 (CIAP1), the cellular inhibitor of apoptosis protein-1 (CIAP2), the X-linked inhibitor of apoptosis protein (XIAP), and B-cell lymphoma-extra large protein (Bcl-XL), [74]. Of equal importance are efforts to identify cellular targets modified by ajoene. Kaschula and colleagues reported on the total synthesis of two fluorescently tagged ajoene probes. These probes allowing researchers to track the movement and localization of ajoene in cancer cells. Both a dansyl-tagged ajoene (DP; Figure 4D) and a fluorescein-tagged ajoene (FOX; Figure 4E) were used to study the localizes of ajoene in mammalian breast-cancer cells [75]. Dansyl ajoene, S-thiolates cellular proteins, and the use of this compound reportedly allowed for the identification of vimentin as a cellular target prone to ajoene attack [76]. 

### 3.3. Cysteine Analogs

**Figure 5:**
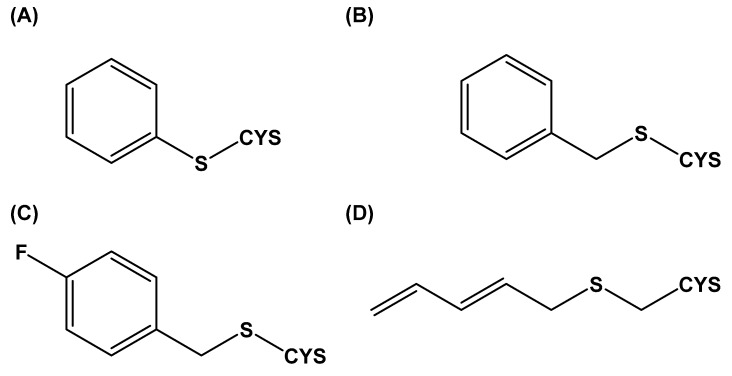
Synthetic thioallyl derivatives described in the work of Pinto [77]. (**A**) Phenyl-S-cysteine, (**B**) benzyl-S-cysteine, (**C**) *para*-fluorobenzyl-*S*-cysteine, and (**D**) penta-1,3-dienyl-*S*-cysteine.

Almost 20 years ago, Pinto and colleagues described the cytotoxic properties of a series of synthetic *S*-cysteinyl compounds that resemble garlic constituents (Figure 5A–D). In this work, the anti-proliferative effects of these compounds on human prostate carcinoma (LNCaP) cells were reported [77]. 

**Figure 6:**
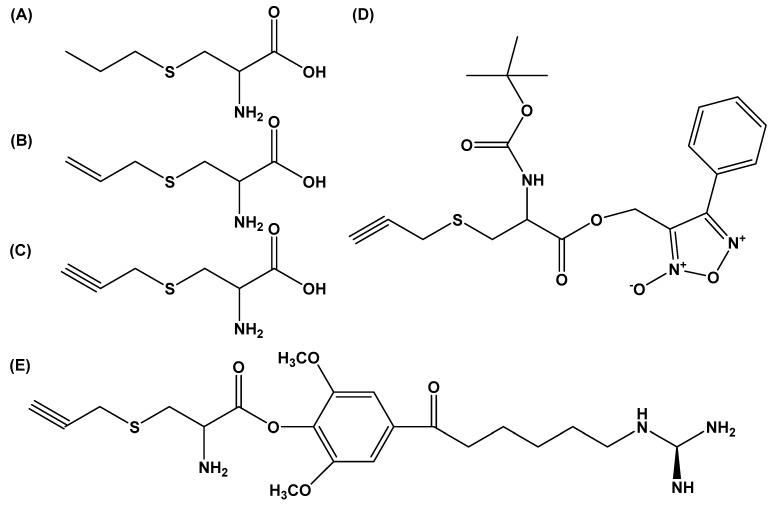
(**A**) *S*-propylcysteine, (**B**) *S*-allylcysteine, (**C**) *S*-propargylcysteine, three widely characterized cysteine analogs. (**D**) Dual H_2_S and NO hybrid molecule ZYZ803. (**E**) Leonurine–SPRC conjugate, 3,5-dimethoxy-4-(2-amino-3-prop-2-ynylsulfanyl-propionyl)-benzoic acid 4-guanidino-butyl ester. This molecule has cardioprotective effect against hypoxia-induced neonatal rat ventricular myocyte damage [80].

In the last decade, one of the most widely characterized cysteine analogs is *S*-propylargyl cysteine (SPRC; Figure 6) identified after an initial screening of several analogs of *S*-allylcysteine [78,79]. 

The pharmacological effects of SPRC are associated with its cardioprotective and proangiogenic properties as determined in several ischemic heart models. Studies show that SPRC is neuroprotective when tested in a model of Alzheimer’s disease [81,82], has anticancer effects [83,84], and is anti-inflammatory [85]. In the cardiovascular system SPRC reduces infarct size, improves cardiac function, and mitigates the production and damaging effects of oxidative stress. These effects are associated with the induction of antioxidant defenses in cardiac tissue, including catalase and superoxide dismutase, and changes in the circulatory levels of H_2_S gas via the upregulation of H_2_S biosynthetic enzyme cystathionine-gamma-lyase (CSE), [82]. Additional research has confirmed the cardioprotective effects of SPRC in animal models of heart failure [86], myocardial infarction [87], doxorubicin-induced cardiotoxicity [88], hypoxic stress [89], and glucose-mediated damage [90]. When tested on primary human umbilical vein endothelial cells, SPRC was proangiogenic via the activation of STAT3 signaling [91]. The anti-inflammatory effects of SPRC were linked to the inhibition of TNF-α-induced ROS production and JNK1/2/NF-κB activation in cells, and reduced the expression of adhesion proteins ICAM and VCAM in endothelial cells. In animal models, SPRC reduced inflammation in models of acute pancreatitis in mice [92], alleviated inflammation anemia [93], and had anti-inflammatory effects in a rodent model of rheumatoid arthritis by modulating Nrf2-ARE signaling [94]. Moreover, in heart failure, SPRC preserves mitochondrial dysfunction through S-sulfhydration of Ca^2+^/calmodulin-dependent protein kinase II (CAMKII) [95]. The same group recently developed a novel SPRC conjugate, designated ZYZ-803, that functions as a combined H_2_S–NO-releasing molecule [96] (Figure 6D). ZYZ-803 contains the H_2_S-releasing moiety of S-propargylcysteine (SPRC) combined with the NO-releasing group of furoxan. Characterization of this ‘gas’ donor revealed that ZYZ-803 can time- and dose-dependently relax the sustained contraction induced by phenylephrine in rat aortic rings, and has 1.5- to 100-fold greater efficacy than that of furoxan or SPRC alone. Additional studies indicated that ZYZ-803 can stimulate endothelial cell angiogenesis [97] via induction of STAT3 signaling and the activation of CAMKII [98]. ZYZ-803 can improve left ventricular remodeling and preserve left ventricular function in isoprenaline-induced heart failure in mice [99]. These protective effects corresponded with the upregulation of CSE, an enzyme important for H_2_S generation, and the enzyme endothelial NO synthase (eNOS) needed for NO production in cardiac tissue. Various endogenous antioxidant proteins, like glutathione peroxidase (GPx) and heme oxygenase 1 (HO-1), also showed increased expression in tissue. Lastly, ZYZ-803 treatment in animals mitigates endoplasmic reticulum stress-related necroptosis following acute myocardial infarction [100].

### 3.4. Sulfide Species

**Figure 7:**
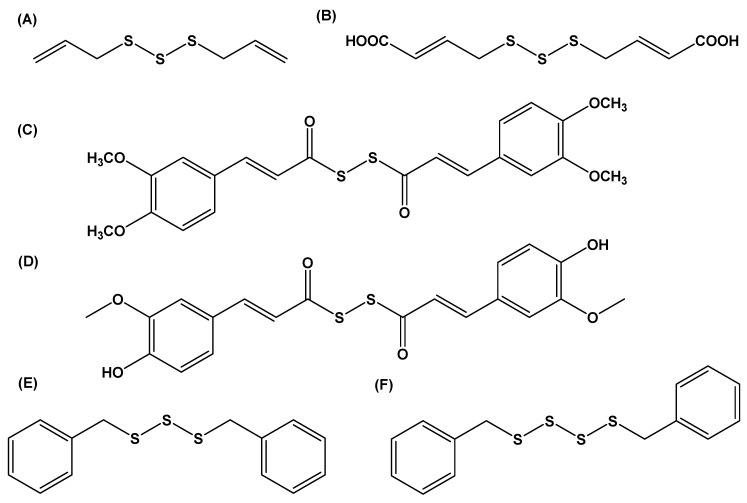
In recent years, interest in (**A**) DATS led to the design and synthesis of several novel sulfide derivatives including (**B**) novel antibacterial agent (2*E*,2*E*)-4, 4-trisulfanediylbis(but-2-enoic acid), and DADS derivatives, (**C**) *bis*[2-(3,4-dimethoxy phenyl) ethenyl] dithioperoxy anhydride and (**D**) *bis*[2-(3-methoxy,4-hydroxy phenyl) ethenyl] dithioperoxy anhydride. These two molecules are templates for developing new multifunctional therapeutics for Alzheimer’s disease treatment [114]. Each compound inhibits Aβ1-42-induced neuronal cell death in human neuroblastoma SH-SY5Y cells. Novel H_2_S-releasing benzyl polysulfides (**E**) benzyl trisulfide and (**F**) benzyl tetrasulfide.

Sulfides are common in many allium preparations, and compounds such as DADS, DATS, and allyl methyl trisulfide (AMS) can occur in appreciable amounts in oils produced during steam distillation. Garlic essential oils contain high amount of sulfur compounds like diallyl trisulfide (37.3–45.9%), diallyl disulfide (17.5–35.6%), and methyl allyl trisulfide (7.7–10.4%) [101,102] (Figure 7A). Sadly, like other allium-derived sulfur compounds, many sulfides are chemically reactive and prone to decomposition, and this limits their use as viable therapeutic agents. Attention has been focused on the screening of sulfide analogs as anti-proliferative agents in cancer cells [103], as inhibitors of HMG–CoA reductase, which is a key enzyme in lipid metabolism [104,105], and as antimicrobial agents [106], (Figure 7B). Research by Saini and colleagues identified *bis*[3-(3-fluorophenyl) prop-2-ene]disulphide from an initial assessment of several synthetic DADs analogs. *Bis*[3-(3-fluorophenyl) prop-2-ene]disulfide induces ROS generation, loss in mitochondrial membrane potential, and changes in the expression of Bcl-2/Bax ratio, leading to the induction of apoptosis in human pancreatic cancer MIA PaCa-2 cells [107]. This compound caused G2/M phase cell-cycle arrest, and the upregulation of p-Chk1 and of inactive phosphorylated Cdc25C protein. Similarly, other synthetic sulfide derivatives appear to be anti-proliferative in cancer cells, suggesting some commonality in their anti-proliferative effects. This property appears to correspond with the induction of ROS production in cancer cells [108,109] and the *S*-thiolation of intracellular proteins [110]. Other pronounced biological effects of synthetic sulfide derivatives include neuroprotection by *bis*[2-(3,4-dimethoxy phenyl) ethenyl] dithioperoxy anhydride and *bis*[2-(3-methoxy,4-hydroxy phenyl) ethenyl] dithioperoxy anhydride derivatives (Figure 7C,D) [111,112], and cardioprotective properties [113]. In recent times, interest in the development of H_2_S-releasing therapeutics has increased. As mentioned, compounds like DATS can generate H_2_S gas, and this property is partly responsible for the biological effects attributed to dietary foods containing this class of molecule [37,114,115,116]. H_2_S is the third gaseous signaling molecule to be characterized, and it has a range of important biochemical and physiological roles in mammals [35,117]. With this in mind, several research groups have focused efforts on the development of synthesis of H_2_S-releasing sulfides, particularly polysulfide species [118]. This work afforded the synthesis of tetrasulfide species in high yields with little need for extensive purification. Several *bis*(aryl) tetrasulfides and *bis*(alkyl) tetrasulfides, including *N*-acetylcysteine-derived tetrasulfides, were produced. These approaches provide a convenient route for the production of several H_2_S-releasing molecules. Similar efforts have led to the production of synthetic benzyl polysulfides (ranging from monosulfide to tetrasulfide derivatives) that can release H_2_S in the presence of thiols like cysteine and glutathione (Figure 7E,F). Both benzyl trisulfide and benzyl tetrasulfide also suppress cell proliferation in bEnd.3 cells [119].

## 4. Conclusions

Over the years, researchers have been fascinated with the isolation of functional components present in the tissue of allium plants, and this has led to the characterization of a whole spectrum of biologically active sulfur compounds. Less widely reported is the growing body of work focused on the development of novel therapeutic agents based on naturally occurring sulfur species found in alliums. Currently, this drive has focused on the identification and optimization of synthetic routes in the production of derivatives of ajoene, *S*-allylcysteine, and various sulfide species. This research has led to the production of several new antimicrobial and anticancer agents that are currently evaluated in preclinical cell-culture and animal models. In the future, we anticipate that this field of research will drive developments in the production of newer sulfur therapeutics. 

## Figures and Tables

**Table 1:** Allium and brassica *S*-Alk(en)yl-l-cysteine sulfoxides (adapted from [2]).

Chemical Name	Structure	Species
*S*-Methyl-l-Cysteine Sulfoxide	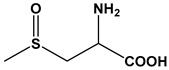	*A. cepa* *A. sativum* *A. chinensis* *Brassica Oleracea*
*S*-Allyl-l-Cysteine Sulfoxide	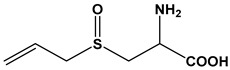	*A. sativum* *A. ursinium* *A. ampeloprasum*
*S*-Propyl-l-Cysteine Sulfoxide	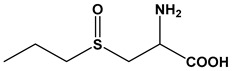	*A. cepa* *A. porrum* *A. fistulosum*
*S*-Propenyl-l-Cysteine Sulfoxide	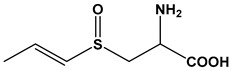	*A. cepa* *A. nutans* *A. schoenoprasum*
*S*-Ethyl-l-Cysteine Sulfoxide	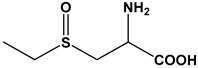	*A. aflatunense* *A. ampeloprasum* *A. victorialis*
*S*-*n*-Butyl-l-Cysteine Sulfoxide	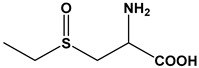	*A. siculum*

## Supplementary Materials

Supplementary materials are available online at https://www.mdpi.com/1420-3049/24/21/4006/s1.
