# Supplementary material for: An Appraisal of Developments in Allium Sulfur Chemistry: Expanding the Pharmacopeia of Garlic
Source: Molecules. 2019 Nov 5;24(21):4006. doi: 10.3390/molecules24214006 (PMC6864437; doi:10.3390/molecules24214006)
Supplement: Supplementary file 1 [file molecules-24-04006-s001.pdf]

Compound	Model	Effect	Reference
**ASCO/thiosulfinate analogues**	Human platelet aggregation induced by either collagen or ADP.	Alliin analogues tested for their inhibitory activity on platelet aggregation. S-oxodiallyl disulphide, found to have a strong inhibitory effect comparable to that of alliin. Other tested compounds do not show any inhibitory effect even at concentrations of 10^{-3}M	50
	Human adenocarcinoma breast cancer cells, MCF-7 cells	Stabilized allicin derivatives on human breast cancer cells in vitro. In this study, a total of 22 stabilized thiosulfinate derivatives. Greater anti-proliferative activity against MCF-7/Dx cells than allicin.	60
	10-day old chick embryos/ Female C56/BL mice/human platelets	All tested organosulfur compounds demonstrated effective inhibition of either FGF or VEG-mediated angiogenesis (anti-angiogenesis activity) in the chick chorioallantoic membrane (CAM) or the mouse Matrigel® models. Allicin and difluoroallicin showed an effective antiplatelet effect. Difluoroallicin showed concentration-dependent inhibition of clot strength compared to allicin and the other organosulfur compounds tested.	61
**Ajoene analogues**	*P. aeruginosa*	The effect of synthetic ajoene toward *P. aeruginosa* were assessed and DNA microarray studies used to reveal a concentration-dependent attenuation QS-controlled virulence factors, including rhamnolipid	68
	P. aeruginosa	25 disulfide bond-containing analogues were synthesized and tested for QS inhibition activities. Reduce QS-regulated virulence factors (elastase, rhamnolipid, and pyocyanin) and successfully inhibit *P. aeruginosa* infection in murine model of implant-associated infection	69
	CT-1 transformed fibroblast cells	Anti-cancer activity of a series of 13 ajoene analogues.	70
	Breast epithelial, MDA-MB-231, SiHa cells; Cervical epithelial HeLa, Oesophageal epithelial, WHCO1, Kyse520, CT1	A small library of ajoene analogues were tested leading to the identification of p-methoxybenzyl derivatives. *In vitro* anti-proliferation activity of p-methoxybenzyl derivatives were tested in six cancer cell-lines and were compared with Z- and E-ajoene. p-methoxybenzyl analogues had enhancement activity of up to twelvefold	71
	WHCO1 oesophageal cancer cells	G(2)/M cell cycle arrest and apoptosis. anti-proliferation activity against WHCO1 oesophageal cancer cells has identified a derivative containing p-methoxybenzyl (PMB)-substituted end groups that is twelve times more active than Z-ajoene, with an IC_{50} of 2.1μM	72
	Oesophageal cancer cell lines WHCO1 and WHCO6, KYSE30 and Het-1A cell lines	DNA microarray analysis of oesophageal cells exposed to ajoene analogues. protein processing in the endoplasmic reticulum (ER) and the unfolded protein response. ER stress sensor GRP78 and induced expression of the ER stress marker CHOP/GADD153. Induction of MAPK proteins, JNK and ERK1/2.	73

	MDR cancer cells (KBV20C and MES-SA/DX5	The anticancer effects of SPA3015, a synthetic ajoene analogue, in P-gp-overexpressing cancer cells. SPA3015 reduced cell viabilities of both KBV20C and MES-SA/DX5 cells and selectively suppressed NF-κB reporter gene activity, and the expression of NF-κB target genes such as CIAP1, CIAP2, XIAP, and Bcl-XL	74
	Breast epithelial, MDA-MB-231; Cervical epithelial HeLa	Dansyl-ajoene was used to identified molecular targets prone to ajoene. The 57 kDa protein vimentin was identified. Ajoene and dansyl-ajoene covalently bind to recombinant vimentin via a disulfide linkage at Cys-328. In cells, non-cytotoxic concentration of ajoene promotes vimentin condensation; and to increase vimentin protein expression. Ajoene inhibited the invasion and migration of both cell types	75-76
**Cysteine analogues**	LNCaP prostate cancer cells	Synthetic S-cysteinyl analogues revealed that growth inhibition was most effective with compounds containing a disulfide or an active diallyl moiety. S-allylmercaptocysteine and S-allylcysteine caused an increase in LNCaP cell reduced glutathione concentrations. Putrescine and spermine concentrations decreased and spermidine increased	77
	Rat acute myocardial infarction (MI) and in vitro hypoxic cardiomyocytes models	Reduction in infarct size, decreased plasma enzymes and reduced malondialdehyde levels when compared to the MI vehicle group. Loss of cell viability in hypoxic cardiomyocytes could be rescued by SPRC mRNA and protein expression of CSE were upregulated by SPRC.	78
	Neonatal rat ventricular myocytes	Synthesis and biological evaluation of novel leonurine-SPRC conjugate, 3,5-dimethoxy-4-(2-amino-3-prop-2-ynylsulfanyl-propionyl)-benzoic acid 4-guanidino-butyl ester. Pharmacological evaluation has shown that 1 possesses potent cardioprotective effect against hypoxia-induced neonatal rat ventricular myocytes damage	79
	Rat, *Rattus norvegicus*	S-propyl-l-cysteine (SPC), S-allyl-l-cysteine (SAC), and S-propargyl-l-cysteine (SPRC) on H_2S production and antioxidant defences in an acute myocardial infarction (MI) rat model. Preserve SOD and GPx activities and also tissue GSH levels while reducing the formation of the lipid peroxidation product MDA in ventricular tissues	80
	Rat, *Rattus norvegicus*	SPRC prevented a decrease of H_2S levels in rat hippocampus induced by LPS. SPRC inhibited tumour necrosis factor (TNF)-α, TNF-α receptor 1 (TNFR1) and Aβ generation, as well as IκB-α degradation and phospho-transcription factors of the nuclear factor κB p65 (p-NF-κB p65) activation.	81
	Rat, *Rattus norvegicus*	SPRC administration at the doses of 40, 80 mg/kg by intraperitoneal injection inhibited cognitive impairment and neuronal ultrastructure damage induced by intra-cerebroventricular injection of 10 μg of Aβ(25-35). SPRC inhibited the expressions of tumour necrosis factor (TNF)-α, cyclooxygenase-2 (COX-2)	82

		mRNA, extracellular signal-regulated kinase (ERK1/2), and the transcription factor nuclear factor κB (NF-κB).	
	H9c2 cardiac myocytes	SPRC prevented nuclear factor-κB (NF-κB) activation and attenuated LPS-induced mRNA and protein expression of tumor necrosis factor-α (TNF-α), and mRNA expression of intercellular adhesion molecule-1 (ICAM-1) and inducible nitric oxide synthase (iNOS).	85
	Heart failure model in the rat	Cardioprotective effects of a novel controlled release SPRC combination on HF rats. Reduction in infarct size and improvement of cardiac function. Modulated antioxidant defences by preserving levels of GSH, CAT and SOD and reducing CK leakage. In addition, CR-SPRC elevated ratio of Bcl-2/Bax and inhibited activity of caspases to protect against myocardial apoptosis	86
	Rat, *Rattus norvegicus*	Controlled release SPRC significantly reduced infarct size and creatine kinase (CK) and lactate dehydrogenase (LDH) leakage and it preserved cardiac function during myocardial infarction. Preservation of glutathione (GSH), catalase (CAT) and superoxide dismutase (SOD) levels whereas reducing malondialdehyde (MDA) levels.	87
	Rat embryonic ventricular myocardial H9c2 cells; Male 8-week-old C57BL/6 mice	SPRC stimulated the activation of STAT3 via gp130-mediated transduction. tunnel in vitro and in vivo. SPRC inhibits doxorubicin induced loss of cell viability, and restored the expression of gp130/STAT3-regulated downstream genes, inhibited apoptosis and oxidative stress, and antagonized mitochondrial dysfunction and intracellular Ca^{2+} overload	88
	Isolated ventricular myocytes	SPRC improved left ventricular functional recovery and suppressed ($[Ca^{2+}]i$) during hypoxia stage and preserved cell viability	89
	C57BL/6 mice; male Sprague–Dawley (SD) rats; Primary HUVEC	SPRC promoted cell proliferation, adhesion, migration, and tube formation of primary human umbilical vein endothelial cells (HUVEC) and increased angiogenesis in the rat aortic ring and Matrigel plug models. Activation of signal transducer and activator of transcription 3	91
	Mice, *Mus musculus*	Significant reduction of inflammation, both in pancreas and lung by SPRC given 3 h prior to the induction of acute pancreatitis. Reduced plasma levels of IL1-beta, IL-1 and increased IL-10.	92
	Eight-week-old male C57BL/6 mice	SPRC reduced serum hepcidin, improved transferrin saturation, and maintained erythrocyte membrane integrity in a chronic mouse AI model. Splenomegaly was ameliorated and splenic iron accumulation reduced. IL-6 content and hepatic Il-6 mRNA were decreased by SPRC, in parallel with reduced hepatic JAK2/STAT3 activation	93
	Male Sprague-Dawley (SD) rats; FLS MH7A cell line.	SPRC attenuated inflammatory mediator expression, reactive oxidase species generation, and the expression and activity of matrix metalloproteinases (MMP)-9 in interleukin (IL)-1β stimulated human rheumatoid fibroblast-like synoviocytes	94

		MH7A. SPRC blocked IL-1β-mediated migration and invasion of MH7A cells. SPRC induced heme oxygenase-1 expression associated with the degradation of Kelch-like ECH-associated protein 1 (Keap1) and nuclear translocation of nuclear factor erythroid 2-related factor 2 (Nrf2).	
	Rat aortic rings	ZYZ-803 could time- and dose-dependently relax the sustained contraction induced by PE in rat aortic rings, with potencies of 1.5- to 100-fold greater than that of furoxan and SPRC. ZYZ-803 increased cGMP level and the activity of vasodilator stimulated phosphoprotein (VASP) in aortic rings	96
	Rat aortic rings, Matrigel plug and murine ischemic hind limb models	ZYZ-803 promotes increased expression of cystathionine γ-lyase (CSE) and endothelial NO synthase (eNOS). Sirtuin-1 (SIRT1), CSE, and/or eNOS small interfering RNA (siRNA) treatment suppressed the angiogenic effect of ZYZ-803.	97
	Mice, *Mus musculus*	ZYZ-803 (1 μM) significantly increased the phosphorylation of STAT3 (Tyr705) and CaMKII (Thr286) in human umbilical vein endothelial cells (HUVECs). STAT3 and CaMKII functioned as positive regulators in ZYZ-803-induced endothelial angiogenesis and STAT3 was important in ZYZ-803-induced CaMKII activation.	98
	Male C57BL6/J mice	ZYZ-803 dose dependently improved left ventricular remodeling and preserved left ventricular function in the setting of isoprenaline-induced heart failure. ZYZ-803 stimulated the expression of cystathionine γ-lyase (CSE) for H_2S generation and the activity of endothelial NO synthase (eNOS) for NO production	99
	Male C57BL/6 mice	In animals, ZYZ-803 preserves cardiac function and reduces infarct size. ZYZ-803 relieves ERS and necroptosis in an AMI heart. In vitro, ZYZ-803 ameliorates ERS-related necroptosis induced by tunicamycin, and such effect has been depending on the receptor-interacting protein 3- (RIP3-) Ca^{2+}-calmodulin-dependent protein kinase (CaMKII) signaling pathway	100
**Sulfide Analogues**	Isolated rat hepatocytes	DADS analogues were found to be less cytotoxic than several statins, such as Lovastatin (IC_{50} = 90 μM), Atorvastatin (125 μM), Simvastatin (>100 μM) in primary rat hepatocyte culture.	103
	Hypercholesterolemia in rats; cholesterol rich diet (%)/2 Wks	Hypolipidemic activities were examined in hypercholesterolemic Wistar rats. Lowered hepatic cholesterol levels by between 13- 37%. Inhibited the activity of HMG-CoA reductase by between 24-56%.	104
	Hypercholesterolemia in rats; cholesterol rich diet (%)/2 Wks	Reduced total lipid levels and decreased 3-hydroxy-3-methylglutaryl-CoA reductase (HMGR) activity. DADS analogues inhibited the activation of sterol regulatory element-binding protein-2 (SREBP-2) and interfered with DNA binding activity of cAMP response element-binding protein (CREB) but not nuclear factor-Y (NF-Y), with upstream regulatory sequences of HMGR	105
	Staphylococcus aureus	A synthetic derivative of DATS namely, (2E, 2E)-4,4-trisulfanediylbis(but-2-enoic acid) (TSDB), exerted a strong inhibitory effect against *Staphylococcus*	106

		aureus, with minimal inhibitory and minimal bactericidal concentrations of 16 and 128 μg/mL. TSDB altered the integrity of *S. aureus* cell membrane but weakly damaged the bacterial cell wall	
	HepG2, PC-3 and HCT-116	4-substituted benzyl analogues of DADS and their selenium counterparts. Anti-proliferative activities and ROS production	108
	Macrophages	Effects of production of reactive oxygen species including superoxide anion ($O_2^{-\cdot}$) , hydrogen peroxide (H_2O_2) and hydroxyl radical (OH·) in phorbol myristate acetate stimulated peritoneal macrophages	109
	WHCO1 oesophageal cancer cells	Synthesis and evaluation of a series of R-propyl disulphide and R-thiosulfonate compounds. Assessed for thiolysis and ROS generation in the cytotoxicity in cancer cells.	110
	SH-SYSY human neuroblastoma cells; Wistar rats.	Anti-Aβ aggregation activity, considerable acetylcholinesterase (AChE) inhibition, high selectivity towards AChE over butyrylcholinesterase (BuChE), potential antioxidant and metal chelating activities	111
	L-NAME induced hypertension in Wistar rats	Reduced the elevated systolic blood pressure (SBP) and the activity of angiotensin converting enzyme (ACE). Preserved nitrite/nitrate (NOx) concentrations and cyclic guanosine monophosphate (cGMP) levels.	112
